# The use of machine learning to understand the relationship between IgE to specific allergens and asthma

**DOI:** 10.1371/journal.pmed.1002696

**Published:** 2018-11-20

**Authors:** Thomas A. E. Platts-Mills, Matthew Perzanowski

**Affiliations:** 1 University of Virginia, Charlottesville, Virginia, United States of America; 2 Columbia University Mailman School of Public Health, New York, New York, United States of America

## Abstract

Thomas Platts-Mills and Matthew Perzanowski provide their expert Perspective on a translational study from Custovic and colleagues that identifies pairings of IgE that show value in estimating risk of concurrent asthma.

In a study recently published in *PLOS Medicine*, Custovic and colleagues [1] report an elegant analysis of the results of specific Immunoglobulin E (IgE) assays on sera from a birth cohort that they have studied in detail over the last 15 years. The IgE assays used a microchip with >100 purified allergens, and their machine learning approach used a hypothesis-free statistical approach to group children with similar allergen sensitization profiles. The primary objective was to evaluate the relationship of the results to the diagnosis of current asthma.

The goal of employing a “machine learning” approach is both as a form of data reduction to avoid spuriously identified associations resulting from multiple comparisons and to identify biologically relevant groupings that may not be recognized with conventional approaches. The use of “hypothesis free” clustering methods with individual allergen components in itself is not novel [2,3]. However, the authors have employed a new network analysis method, which is improved over previous methods because it can capture nonlinear relationships and does not rely on assumption of a parametric probability. As the authors show, this approach can lead to improved sensitivity and specificity. With this model, they were able to show that pairings of allergens (e.g., the cat allergen Fel d 1 and the peanut allergen Ara h 1) were more predictive of the child having asthma than individual components, although we must be cautious in the generalizability of the specific patterns identified, in this single cohort in the United Kingdom, because the pairing may represent host susceptibility to making an IgE response or common co-exposures, which could be allergens or adjuvants. Still, the concept of complicated connections between individual allergenic proteins and allergic disease are likely to apply more broadly and are important to consider in future studies.

In vitro assays for IgE go back to 1967, when the Radioallergosorbent test (RAST) was published within a year of the discovery of IgE. [4–6] The great-grandchild of RAST is the current most widely recognized assay for IgE, which uses a high-capacity sponge, can be used either with an allergen extract or a purified allergen, and can bind micrograms of protein. In contrast to the “singleplex” assays, microchip assays have been developed to carry large numbers of purified proteins. The ISAC chip used in the current study has purified proteins on spots with nanogram quantities. The microchip gives results in standardized units that are similar to, but not the same as, the absolute units where one unit is equal to 2.4 ng of IgE. Overall, the microchip is less sensitive and can definitely be blocked by the presence of high-titer IgG antibodies in the serum. This is a real problem in the evaluation of IgE to milk proteins in eosinophilic esophagitis but is unlikely to be relevant in relation to asthma in childhood [7]. In relation to asthma, only occasional allergens will give rise to IgG antibodies sufficient to inhibit the microchip results. However, immunotherapy can certainly achieve high-titer IgG antibodies. The inevitable result of having 100 proteins is that many of them are both structurally similar and also immunologically cross-reactive. An excellent example is the lipocalins, in that the horse protein Equ c 1 can appear to be a dominant allergen when the primary exposure is to the cross-reactive dog allergen Can f 1 [8]. Equally, the pathogenesis-related proteins cross-react extensively, which means that many patients who are primarily allergic to birch pollen (Bet v 1) experience oral symptoms from eating apples (Mal d 1) or hazelnuts (Cor a 1). However, of these, only Bet v 1 has been associated with asthma.

The authors point out that some asthma guidelines do not recommend allergy testing, and they argue that better understanding of the relationship of specific allergen IgE responses to asthma is necessary. They certainly demonstrate that their analysis of the microchip assay results in a striking prediction of current asthma diagnosis in the birth cohort. However, skin testing or IgE assays usually occurs in children when the diagnosis is already clear or very likely from history and examination. The primary objective at that point is to inform the physician, to help refine the diagnosis, and to educate the patient. Of these, educating the patients may be the most important. Two years ago, this same group of investigators in the UK published an important study showing that measures to reduce house dust mite allergens in the home could decrease the risk of acute asthma episodes in childhood [9]. Equally, a major inner city study demonstrated a significant effect of allergen avoidance in asthma [10]. In each case, the measures recommended were based on a specific diagnosis of sensitization. Furthermore, there is increased evidence that the titer of IgE antibodies is an important predictor of the persistence of asthma, and also of the probability of an acute episode of asthma following a rhinovirus infection [8,11]. In the analysis reported by Custovic and colleagues, one *Alternaria* allergen was included in their Fig 3, but this allergen, Alt a 1, does not appear in the cluster analysis in their Table 1, and no *Aspergillus* allergens are included. The implication is that no fungal allergens are relevant to asthma at this age. In other analyses at the same age, *Aspergillus* sensitization—though not common—was shown to have the strongest association with asthma [12]. In older patients, *Alternaria* sensitization has often been shown to have a strong relationship to severity [13]. In our practice, an initial screen with 10 allergens may indicate the need for more focused evaluation of fungi, or even foods. Although the analysis presented argues cogently that the data can provide better information to help with diagnosis, both the quantity of data and the details of the analysis would not be helpful in educating either patients or their doctors. The microchip assays have the potential to provide extensive information about a wide range of allergic disease using a modest quantity of serum, approximately 80 μL. The authors have shown that careful analysis of the results can provide dramatically enhanced data on the relevance of the results to a major allergic disease. The way forward may depend on designing more specific selections of allergens on a microchip and microchips designed for different diseases.

## Abbreviations

IgE: Immunoglobulin E
RAST: Radioallergosorbent test
